# Case Report: Primary ciliary dyskinesia due to CCNO mutations: a Chinese pediatric case series and literature review

**DOI:** 10.3389/fped.2024.1458660

**Published:** 2024-09-24

**Authors:** Lejun Tong, Li Li, Wenjian Wang, Jiehua Chen

**Affiliations:** Department of Respiratory Diseases, Shenzhen Children’s Hospital Affiliated to Shantou University Medical College, Shenzhen, China

**Keywords:** primary ciliary dyskinesia, CCNO gene, genetic diagnosis, diffuse panbronchiolitis, bronchiectasis

## Abstract

Primary ciliary dyskinesia (PCD) is a hereditary disorder characterized by defects in cilia that impair mucociliary clearance. This study focuses on PCD caused by mutations in the Cyclin O (CCNO) gene and reports on three cases involving Chinese children. Case 1 was an 8-year-and-3-month-old boy who presented with respiratory distress after birth and later developed a recurrent productive cough and purulent nasal discharge. He was initially diagnosed with diffuse panbronchiolitis (DPB) due to the presence of diffuse micronodules in lung CT scans. Case 2 was the younger sister of case 1. She also presented with respiratory distress after birth, with a chest radiograph revealing atelectasis. She required oxygen supplementation until the age of 2 months. Case 3 was a 4-year-and-4-month-old girl with a history of neonatal pneumonia, persistent pulmonary atelectasis, and recurrent lower respiratory tract infections. Her chest radiograph also showed diffuse micronodules. In all three cases, the final diagnosis of PCD was confirmed by genetic testing. Cases 1 and 2 exhibited homozygous c.248_252dup TGCCC (p.G85Cfs*11) mutations in the CCNO gene, while case 3 harbored a homozygous c.258_262dup GGCCC (p.Q88Rfs*8) mutation. A literature review indicated that the common clinical features of CCNO-PCD include neonatal respiratory distress (40/49, 81.6%), chronic cough (31/33, 93.9%), rhinosinusitis (30/35, 85.7%), bronchiectasis (26/35, 74.3%), and low nasal nitric oxide (nNO, 40/43, 93.0%). Notably, situs inversus has not been reported. In CCNO-PCD patients, cilia may appear structurally normal but were severely reduced in number or entirely absent. Lung CT scans in these patients may exhibit diffuse micronodules and “tree-in-bud” signs, which can lead to a clinical misdiagnosis of DPB. nNO screening combined with genetic testing is an optimized diagnostic strategy. Treatment options include the use of anti-infective and anti-inflammatory agent, along with daily airway clearance. This study underscores the importance of genetic testing in neonates and children with suspected PCD or those clinically diagnosed with DPB to enable an early diagnosis and prompt intervention, thereby enhancing the prognosis for these patients.

## 1 Introduction

Primary ciliary dyskinesia (PCD) is a genetically and clinically heterogeneous disorder, commonly manifesting as recurrent productive cough, respiratory infections, chronic sinusitis, and otitis media. Approximately half of the patients present with situs inversus totalis (1), which could raise suspicion of PCD if combined with chronic respiratory symptoms and bronchiectasis. To date, over 50 genes associated with PCD have been identified (2), with most mutations resulting in abnormalities in specific ultrastructural components of cilia, thereby impairing ciliary function.

The Cyclin O (CCNO) gene was first reported by Wallmeier et al. (3) in 2014. In this genotype of PCD, situs inversus has not been reported and cilia are significantly reduced or absent, while the ultrastructure of the axoneme may appear normal (3–6). This can lead to missed or delayed diagnosis when relying solely on conventional transmission electron microscopy (TEM) or high-speed videomicroscopy analysis (HSVA). Lung CT scans in patients with CCNO-PCD may exhibit diffuse micronodules and “tree-in-bud” signs, which can be misdiagnosed as diffuse panbronchiolitis (DPB). Despite ongoing reports of new cases, there remain a limited number of documented cases globally.

In this article, we report three cases of PCD caused by CCNO gene mutations and review the literature to elucidate the clinical features and diagnostic challenges and promote early recognition of this PCD genotype.

## 2 Case presentation

### 2.1 Case 1

**Figure 1:**
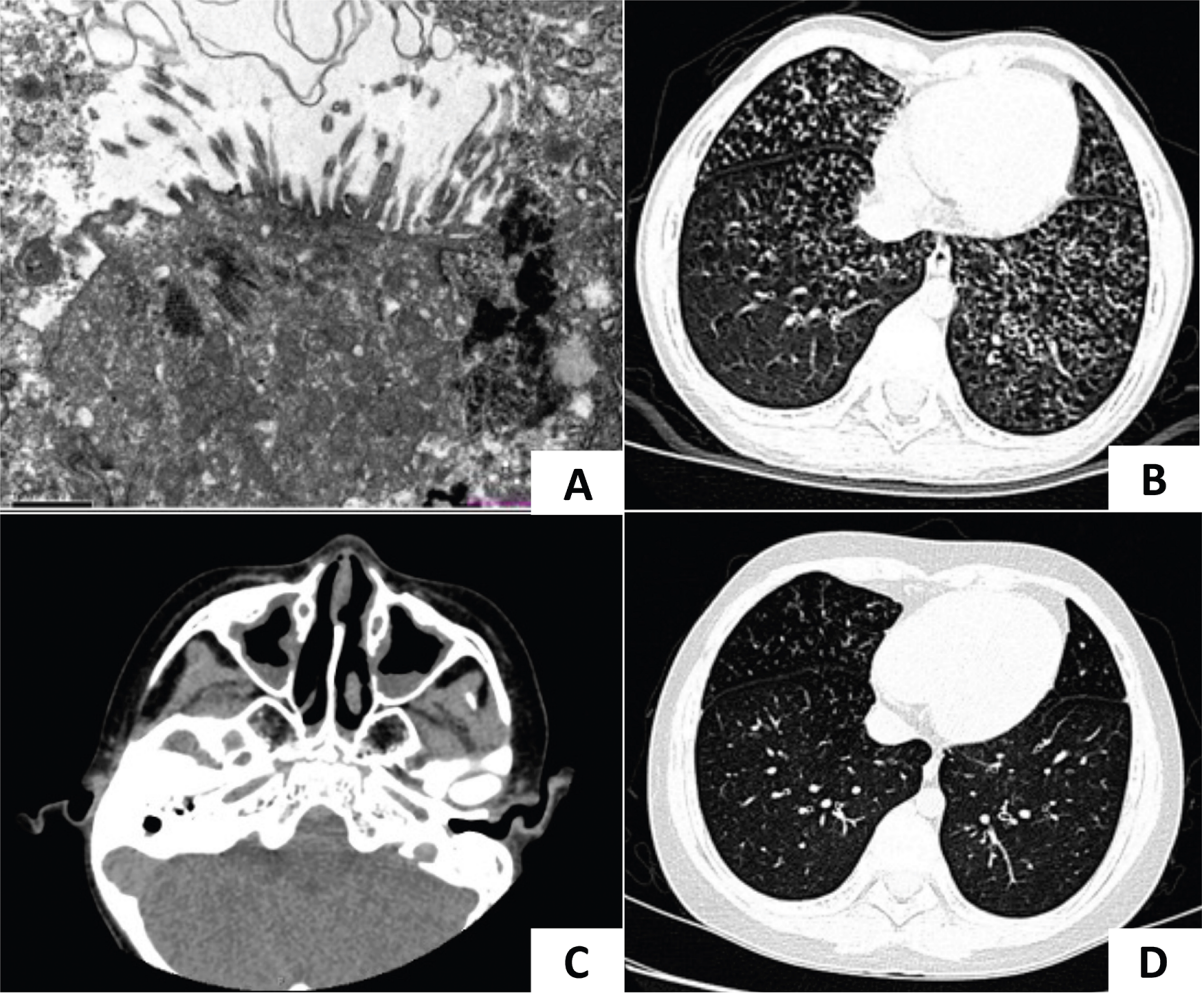
CT imaging and TEM of case 1. **(A)** TEM of case 1 showed microvilli on the epithelial surface, inflammatory cell infiltration, and absent cilia in multiple sections. **(B)** A lung CT scan identified bilateral diffuse micronodules and “tree-in-bud” signs. **(C)** A sinus CT scan showed mucosal thickening of bilateral nasal cavities and maxillary sinuses. **(D)** Lesions in the lower lungs improved after treatment.

An 8-year-and-3-month-old boy was admitted to the respiratory department of our hospital with a 6-day history of cough and fever. Born at term, the boy developed tachypnea 12 h after birth, which was diagnosed as neonatal pneumonia. He had no history of mechanical ventilation or prolonged oxygen supplementation and exhibited situs solitus. Since he was 1 year old, he had experienced a recurrent productive cough and nasal discharge, with a temporary improvement following antibiotic treatment. He showed no hydrocephalus or hearing problems. His cognitive, motor, and language development were normal. At 7 years of age, he was admitted to our hospital for an acute respiratory exacerbation. A chest CT scan revealed diffuse small nodules in the lower fields of both lungs and bronchiectasis. Bronchoscopy indicated suppurative bronchitis. A bacterial culture of bronchoalveolar lavage fluid (BALF) identified *Haemophilus influenzae*. Immunological tests, including T and B lymphocyte counts, serum immunoglobulin levels, and the respiratory burst test, were unremarkable. His levels of specific immunoglobulin E (IgE) for food and inhalant allergens, as well as total IgE levels, were within normal range. TEM revealed an absence of cilia in multiple sections (Figure 1A, KingMed Diagnostics, Guangzhou, China). His nasal nitric oxide (nNO) levels were consistently measured at 4 parts per billion (ppb) across multiple assessments. His fractional exhaled nitric oxide (FeNO) levels were recorded at 2 and 4 ppb in repeated measurements (using the SUNVOU nitric oxide device, Wuxi, China, with a flow rate of 10 ml/s for nNO and 50 ml/s for FeNO).

On physical examination, the patient exhibited no dyspnea or cyanosis. Auscultation revealed coarse to moderate crackles in both lungs, with diminished breath sounds in the left lung.

Auxiliary examination: A lung CT scan demonstrated diffuse centrilobular micronodules in the left lingula segment, left lower lobe, and right middle and lower lobes. Bronchial wall thickening and lumen dilation were also observed (Figure 1B). A sinus CT scan revealed a thickening of the mucosa in the nasal cavity and the maxillary sinus (Figure 1C). A pulmonary function test indicated mild obstructive ventilatory impairment, with forced vital capacity (FVC) at 90.46% of the predicted value, forced expiratory capacity in 1 s (FEV_1_) at 81.27% of the predicted value, and an FEV_1_/FVC ratio at 75.51% of the predicted value. Bronchoscopy revealed inflammation of the bronchial mucosa. Bacterial culture, *Mycoplasma pneumoniae* DNA, and *Mycobacterium tuberculosis* DNA testing of BALF were negative. nNO was measured at 12 ppb (7.2 nl/min).

Diagnosis and treatment: Given the patient's history of neonatal respiratory distress, chronic respiratory symptoms, bronchiectasis, and markedly decreased nNO levels, PCD was considered. However, the previous TEM results showed absent cilia and the lung CT scan revealed diffuse centrilobular micronodules rather than the typical consolidation, atelectasis, or significant bronchiectasis commonly associated with PCD, leading to an inconclusive diagnosis. According to the Japanese diagnostic criteria for DPB (7), which include

1. Persistent cough, sputum, and exertional dyspnea;
2. History of chronic paranasal sinusitis;
3. Bilateral diffuse small nodular shadows on a plain chest radiography film or centrilobular micronodules on chest computed tomography images;
4. Coarse crackles;
5. FEV_1_/FVC <70% and PaO2 <80 mmHg; and
6. Titer of cold hemagglutinin ≥64.

The first four criteria were met in case 1, raising suspicion of DPB. The patient was treated with intravenous amoxicillin–sulbactam, low-dose oral erythromycin, a nasal glucocorticoid, and expectorants.

Follow-up: A review of the chest CT scan 1 year after discharge showed persistent diffuse centrilobular micronodules. Whole exome sequencing (WES, MyGenostics, Beijing, China) identified a homozygous c.248_252dup TGCCC (p.G85Cfs*11) mutation in the CCNO gene, confirming a definitive diagnosis of PCD. Following regular daily treatment with hypertonic saline nebulization (3%), airway clearance, and low-dose oral erythromycin, his productive cough and lung CT improved (Figure 1D). A pulmonary function test conducted at 13 years of age revealed normal ventilatory function, but diffuse micronodules persisted in the lower fields of both lungs.

### 2.2 Case 2

**Figure 2:**
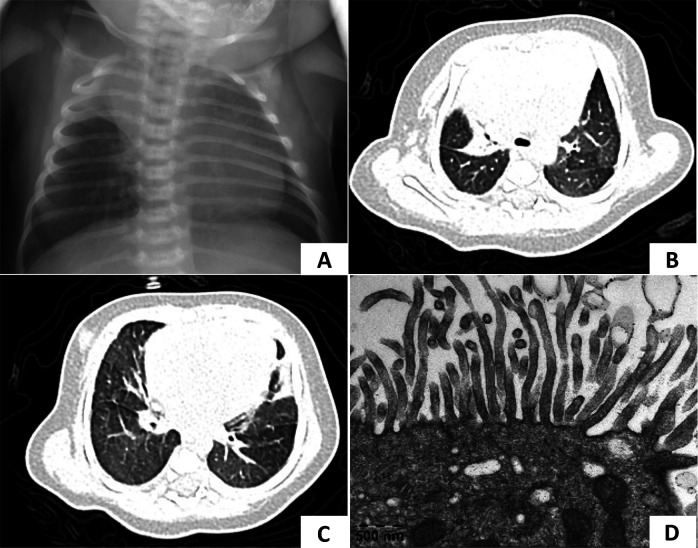
Chest radiography and TEM of case 2. **(A)** Chest x-ray showed atelectasis of the right upper lobe at 1 week after birth. **(B,C)** At 1 month of age, a lung CT scan showed a decreased volume of the right upper lobe and patchy opacities in both lungs which demonstrated pneumonia with right upper lobe atelectasis. **(D)** TEM showed microvilli with few cilia.

A female infant was admitted to the neonatal department of our hospital 12 h after birth. She was the younger sister of case 1, born at term, and exhibited grunting and tachypnea 8 h postnatally. On physical examination, crackles were auscultated in both lungs. A chest x-ray revealed atelectasis of the right upper lobe (Figure 2A). The patient exhibited situs solitus. A nasopharyngeal swab for Group B streptococcus DNA was negative. Multiplex polymerase chain reaction (PCR) tests for a panel of respiratory pathogens, including metapneumovirus, adenovirus, rhinovirus, parainfluenza virus, influenza B virus, chlamydia, *M. pneumoniae*, influenza A virus, bocavirus, coronavirus, and respiratory syncytial virus, were all negative. Toxoplasmosis, rubella, cytomegalovirus and herpes simplex virus (TORCH) screenings were unremarkable. Echocardiography revealed a patent foramen ovale. Bronchoscopy showed inflammation of the bronchial mucosa and laryngomalacia. The bacteria culture of BALF was negative. High-throughput sequencing of BALF identified *Rothia mucilaginosa* (11,684 sequences) and *Streptococcus mitis* (9,464 sequences), which were considered colonization. Sanger sequencing confirmed the same mutation in this patient as her brother, leading to a diagnosis of PCD. She was treated with heated, humidified high-flow nasal cannula oxygen therapy and intravenous penicillin combined with ceftazidime.

Follow-up: After discharge, the patient continued to require family nasal cannula oxygen supplementation at home. She experienced two episodes of acute respiratory exacerbations at 1 and 5 months of age, respectively. Lung CT scans revealed pneumonia with persistent right upper lobe atelectasis (Figures 2B,C). TEM showed normal microvilli with a few cilia (Figure 2D). With regular daily hypertonic saline nebulization and sputum suction, oxygen supplementation was discontinued by 2 months of age.

### 2.3 Case 3

**Figure 3:**
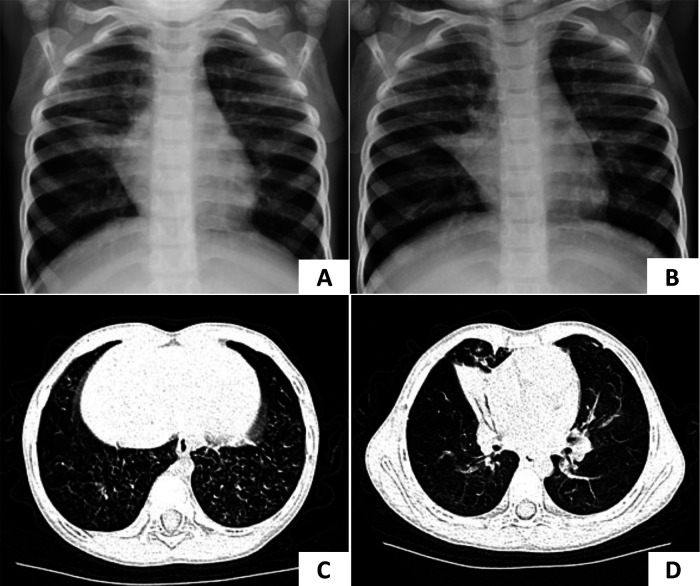
Chest radiography of case 3. **(A,B)** Atelectasis of the right middle lobe was persistent in two separate x-rays. **(C,D)** The lung CT scan showed diffuse micronodules in the lower fields of both lungs and decreased volume of the right middle lobe which were considered pneumonia with atelectasis of the right middle lobe.

A 4-year-and-4-month-old girl was admitted to our hospital with a 2-week history of cough. She had a history of pneumonia and atelectasis during the neonatal period. She required non-invasive ventilation in the neonatal period and continued to require oxygen supplementation until 6 months of age. The patient exhibited situs solitus and had a history of recurrent lower respiratory tract infections (LRTIs) and nasal discharge. A sputum culture identified *H. influenzae*. Bronchoscopy revealed suppurative bronchitis. Multiple chest x-rays showed persistent atelectasis of the right middle lobe (Figures 3A,B). On physical examination, crackles were auscultated in both lungs. Given the history of mechanical ventilation, oxygen supplementation, recurrent LRTIs, and persistent atelectasis, PCD was suspected. WES revealed a homozygous c.258_262dup GGCCC (p.Q88Rfs*8) mutation in the CCNO gene, confirming the diagnosis of PCD. During follow-up, lung CT scans showed diffuse micronodules in the lower fields of the lungs bilaterally, with persistent atelectasis in the right middle lobe (Figures 3C,D).

## 3 Literature review

A literature search was conducted in PubMed using the keywords “primary ciliary dyskinesia”, “immotile-cilia syndrome” and “CCNO gene” to find studies published before April 2024. A total of 15 articles were reviewed, including 1 in Chinese and 14 in English (3, 4, 8–20). In total, including our 3 cases, 58 patients (26 males and 32 females) were included. The demographic and clinical features of these patients are summarized in Table 1, while studies on CCNO-PCD are summarized in Supplementary Table 2.

**Table 1:** Demographic and clinical features of CCNO-PCD.

Items	Number (percentage)
Male	26/58 (44.8%)
Female	32/58 (55.2%)
Neonatal respiratory distress	40/49 (81.6%)
Chronic cough/wet cough	31/33 (93.9%)
Rhinosinusitis	30/35 (85.7%)
Otitis media	17/33 (51.5%)
Conductive hearing loss	1
Infertility	2
Tubbiness thorax	1
Clubbing finger	5
Congenital heart defect	2
Hydrocephalus	1
Diffuse micronodules or tree-in-bud sign in lung CT	7
Situs inversus	0
Atelectasis	10/12 (83.3%)
Bronchiectasis	26/35 (74.3%)
Low nNO^a^	40/43 (93.0%)
Lung transplantation	4
Lobectomy	2
Growth hormone deficiency	1
Selective IgM deficiency	1
TEM	Total: 18
Few or absent cilia	17 (94.4%)
Mislocalized basal bodies and attached rootlets	5 (27.8%)
Normal ciliary ultrastructure	2 (11.1%)
Lack basal bodies	7 (38.9%)
Microtubular disorganization and central pair loss	1 (5.6%)
HSVA	Total: 24
Few or absent cilia	19 (79.2%)
Uncoordinated CBP	3 (12.5%)
Hypokinetic cilia	5 (20.8%)
Normal CBF	2 (8.3%)

CBP, ciliary beat pattern; CBF, ciliary beat frequency.

^a^ Low nNO was determined according to each study if specified, otherwise, it was determined as ≤77 nl/min.

## 4 Discussion

PCD is a highly heterogenous disorder resulting from ciliary dysfunction. To date, over 50 pathogenic genes have been identified (2). Most PCD genotypes lead to defects of specific ciliary ultrastructural components, such as the outer and inner dynamin arms, nexin links, and radial spokes (21), resulting in ciliary immotility or dyskinesia. PCD caused by mutations in the CCNO gene presents with reduced or absent cilia, a phenotype known as ciliary aplasia. However, the ciliary ultrastructure can be preserved (3–6), although a recent report has described microtubular disorganization and central pair loss (20). Residual cilia may beat in a normal pattern but are unable to beat coordinately due to their sparse distribution, ultimately impairing ciliary clearance. Consequently, this phenotype is also referred to as congenital mucociliary clearance disorder with reduced generation of multiple motile cilia (RGMC) (3).

Absent or decreased cilia might initially be mistaken for secondary loss due to brushing or infection. However, a previous study observed a severe reduction in motile cilia and basal bodies in the apical cell region of individuals with CCNO mutations, as well as defects in the migration of cytoplasmic basal bodies (3). An *in vitro* ciliogenesis study confirmed a ciliary generation defect in CCNO-PCD patients, and further research using *Xenopus* embryos demonstrated that CCNO is critical for the amplification and migration of mother centrioles (3). Therefore, the generation defect appears to be the primary cause of the ciliary aplasia phenotype, rather than secondary loss. In addition, DNAH5 and CCDC39, which are essential for ciliary beating, were found to be expressed in the residual cilia (3). These findings help explain the reduced number of cilia with preserved motility in the remaining cilia. Notably, no laterality defects have been reported in CCNO-PCD. This phenomenon has been investigated in mouse models, where it was shown that the ability to form multiple motile cilia is not entirely lost in CCNO-deficient embryos (22). Even as few as two rotating cilia are sufficient to generate nodal flow and establish left–right asymmetry (23), preventing the appearance of laterality defects due to the preservation of residual cilia.

Patients with CCNO-PCD commonly present with neonatal respiratory distress (40/49, 81.6%), chronic cough (31/33, 93.9%), rhinosinusitis (30/35, 85.7%), and otitis media (17/33, 51.5%). However, the clinical presentation and disease progression can be highly variable. For instance, one child was initially misdiagnosed with bronchiolitis obliterans due to recurrent cough and wheezing with exercise intolerance (16), while another was diagnosed with asthma due to recurrent wet cough without a history of sinusitis or otitis, though he did not respond to asthma medications (15). Two children were considered to have coexistent asthma (13). Alhalabi et al. (19) reported two siblings in whom bronchiectasis was detected at 14 years of age in the elder sister and 7 years of age in the younger sister. Similarly, in our case 1, bronchiectasis was identified at 7 years of age. However, the reported younger sibling exhibited diffuse cystic bronchiectasis at this age, whereas our case 1 demonstrated only mild bronchial wall thickening and lumen dilation. Moreover, our case 1 maintained normal pulmonary ventilatory function at 13 years of age, while the reported elder sibling progressed to end-stage lung disease requiring lung transplantation at 17 years old, highlighting the heterogeneity in the disease progression of CCNO-PCD. Previous studies have demonstrated that lung diseases in CCNO-PCD tend to manifest earlier and progress more severely compared to other PCD genotypes. Emiralioğlu et al. (12) demonstrated that patients with CCNO variants showed an early onset of symptoms, with the lowest median age of diagnosis at 3 years old. Raidt et al. (24) found that individuals with CCNO variants had the poorest median FEV_1_ z-score among a cohort of 1,072 genotyped PCD individuals with available lung function data. Extrapulmonary manifestations in CCNO-PCD patients include infertility, arrested hydrocephalus, hearing impairment, and congenital heart defects (3, 4, 9, 12). In addition, one child with a growth hormone deficiency and one with a selective IgM deficiency have been reported (15, 17).

In patients with CCNO-PCD, lung radiographs frequently reveal atelectasis (10/12, 83.3%) and bronchiectasis (26/35, 74.3%). In cases 2 and 3, atelectasis was observed in the same lung field in repeated radiographic tests, suggesting that PCD should be suspected in children with persistent atelectasis. Interestingly, five previously reported cases of CCNO-PCD demonstrated diffuse micronodules and “tree-in-bud” signs in lung CT scans (9, 11, 14, 16, 19), similar to those seen in cases 1 and 3, which mimic DPB. Combined with the chronic respiratory manifestations overlapping with DPB, it is difficult to determine whether these imaging features are pathognomic for DPB, represent a common pattern in CCNO-PCD, or suggest that PCD may be an underlying etiology of DPB, as a previous study found that nNO is also low in patients with DPB (7). Chen et al. (25) reported a PCD case without situs inversus yet complicated with DPB (genetic testing was not available) and reviewed the literature, identifying only 17 reported cases of Kartagener's syndrome complicated with DPB between 1999 and 2014, all of which exhibited situs inversus. Although diffuse bronchiolitis-like changes have been reported in two PCD patients with genetically confirmed CCDC39 and CCDC40 mutations, both of these cases also exhibited situs inversus (26, 27). The relatively frequent occurrence of a diffuse bronchiolitis-like change in CCNO-PCD, as seen in our cases and the reviewed literature, suggests that clinically diagnosed DPB with situs solitus and equivocal ciliary ultrastructural findings should be differentiated from CCNO-PCD, even if the clinical criteria for DPB are met. As genetic diagnosis improves, the genetic bases of various diseases are increasingly illuminated. Whether DPB is an isolated idiopathic inflammatory disease or a clinical syndrome that at least results from CCNO-PCD needs to be further studied. In addition, *H. influenzae* is one of the most common bacteria in airways affected by bronchiectasis (28). In two of our cases that presented with diffuse micronodules in lung CT scans, cultures of respiratory specimens were positive for *H. influenzae*. Okada et al. (29) found that centrilobular nodules appeared in lung CT scans in 64.9% of patients with acute *H. influenzae* pulmonary infection, suggesting that infectious factors may also contribute to the diffuse bronchiolitis-like change observed in these patients.

The diagnosis of PCD relies on a combination of typical history and diagnostic tests. Both the European Respiratory Society (ERS) and the American Thoracic Society (ATS) recommend using nNO as the primary screening tool for cooperative patients with a typical history as nNO levels are consistently low in PCD patients (≤77 nl/min) (30). In our review, nNO was significantly reduced in 93.0% (40/43) of CCNO-PCD patients, with only three cases presenting normal levels, reinforcing its value as an initial screening test for CCNO-PCD. In the subsequent diagnostic program, the ERS guidelines recommend using HSVA to observe ciliary beating frequency or TEM to assess ciliary ultrastructure (21). However, HSVA and TEM may fail to visualize the cilia due to reduced or absent cilia, as observed in 94.4% (17/18) of TEM and 79.2% (19/24) of HSVA assessments. Even if the cilia are observed, the absence of hallmark ciliary ultrastructural defects may lead to an ambiguous or delayed diagnosis. Recent research has demonstrated that the sensitivity of TEM varies depending on the regional prevalence of distinct PCD genotypes. In Turkey, where CCNO and DNAH11 variants are prevalent, TEM failed to diagnose more than half of the PCD cases (24). In China, a previous cohort study has identified CCNO variants as the fifth most prevalent genotype, a finding consistent with a multinational cohort study that included patients from Europe, Asia, and South America (13, 24). Given the relatively high prevalence of CCNO variants, skipping conventional modalities such as TEM and HSVA, nNO screening combined with genetic testing may enhance the detection of PCD and lead to more accurate diagnoses.

The treatment of PCD primarily focuses on managing acute exacerbations caused by infections and ensuring long-term airway clearance, which can compensate for the ciliary clearance function. Effective antibiotic therapy can delay the progression of bronchiectasis. Prophylactic macrolides may be used in patients aged 7 years and older who experience recurrent acute exacerbations. A multinational randomized trial including 90 PCD patients found that maintenance therapy with azithromycin was well tolerated and halved the rate of acute exacerbations (31). Case 1 showed clinical improvement and a significant reduction in small airway lesions in lung CT scans following regular oral low-dose erythromycin and airway clearance with hypertonic saline nebulization. Similarly, Zhang et al. (14) reported a case of CCNO-PCD in which the patient experienced a reduction in diffuse lung nodules after 2 months of oral azithromycin, highlighting the positive effects of macrolides and airway clearance. Surgical interventions are generally reserved for complications. Lobectomy may be considered in cases with significant bronchiectasis. Lung transplantation remains an option for patients with end-stage lung disease to extend survival (32). In the reviewed literature, one child underwent left lower lobectomy at 4 years of age due to persistent atelectasis and recurrent respiratory symptoms. Postoperatively, lower respiratory tract infections decreased, but the patient continued to experience a chronic wet cough and obstructive ventilatory impairment (4). Four patients underwent lung transplantation due to end-stage lung disease at the ages of 34, 34, and 43 years, and one before adulthood (3, 8, 19).

Our study highlights the importance of suspecting CCNO-PCD in term newborns with unexplained later-onset respiratory distress and in children with chronic respiratory symptoms, persistent atelectasis, or bronchiectasis in radiography. No laterality defects have been reported in CCNO-PCD. The clinical manifestations and disease progression are highly heterogeneous, with lung disease appearing earlier and more severely compared to other PCD genotypes. Diffuse micronodules and “tree-in-bud” signs in lung CT scans may lead to a clinical misdiagnosis of DPB. CCNO-PCD may be overlooked if the diagnosis is only based on an airway mucosal biopsy, due to reduced or absent cilia without hallmark ciliary ultrastructural defects. A combination of nNO screening and genetic testing offers an optimized diagnostic approach. In addition, parents of children with PCD should be offered prenatal screening and genetic counseling if planning to have more children.

## Data Availability

The original contributions presented in the study are included in the article/Supplementary Material, further inquiries can be directed to the corresponding author.
