# Supplementary material for: Case Report: Primary ciliary dyskinesia due to CCNO mutations: a Chinese pediatric case series and literature review
Source: Front Pediatr. 2024 Sep 24;12:1458660. doi: 10.3389/fped.2024.1458660 (PMC11458413; doi:10.3389/fped.2024.1458660)
Supplement: Supplementary file 1 [file Table1.pdf]

Supplementary Table 1. Clinical information of case 1, 2 and 3

Case number	Age at diagnosis	Sex	Clinical manifestations & History	Lung radiograph	Echocardiography	nNO	TEM	Genetic test	Follow-up
1	9-year-old	Male	Tachypnea 12 hours after birth, chronic productive cough, chronic sinusitis, crackles, without hydrocephalus or hearing problems, normal cognitive, motor and language development	Bilateral diffuse micronodules, “tree-in-bud” sign, bronchiectasis	Normal	2.4nL/min, 7.2nL/min	Absent cilia in multiple TEM observation	Homozygous c.248_252dup TGCCC (p.G85Cfs*11) mutation in the CCNO gene	Daily 3% hypertonic saline nebulization, low-dose oral erythromycin, lung CT improved, maintained normal ventilatory function at 13 years of age
2	0.5-month-old	Female	Grunting and tachypnea 8 hours after birth, crackles, requiring heated, humidified high-flow nasal cannula oxygen therapy after birth	Bilateral patchy opacity, right upper lobe atelectasis after birth	Patent foramen ovale	N/A	Microvilli with few cilia	Homozygous c.248_252dup TGCCC (p.G85Cfs*11) mutation in the CCNO gene	Family nasal cannula oxygen supplementation until 2 months of age, persistent right upper lobe atelectasis at 1 month of age, daily 3% hypertonic saline nebulization
3	4-year-and-4-month-old	Female	Neonatal pneumonia, non-invasive mechanical ventilation after birth, oxygen supplementation until 6-month-old, crackles, recurrent LRTIs	Persistent atelectasis of right middle lobe, bilateral diffuse micronodules	N/A	N/A	N/A	Homozygous c.258_262dup GGCCC (p.Q88Rfs*8) mutation in the CCNO gene	N/A

nNO levels were measured by SUNVOU nitric oxide device, Wuxi, China (flow rate was 10 milliliters per second);
TEM was performed by KingMed Diagnostics, Guangzhou, China;
Genetic test was performed by MyGenostics, Beijing, China;

Supplementary Table 2. Summary of reported CCNO-PCD cases

Study	Origin	Case number	Clinical information	nNO	TEM	HSVA	Genetic testing
Wallmeier et al. (2014)	4 from Kuwaiti family, others were not specified	16	5 males and 11 females, nRDS (12/16), all were situs solitus, 1 female presented with infertility and used assisted reproduction, 2 underwent lung transplantation at age 34, in 2 families, siblings died in early childhood	Markedly reduced in 11 cases (<100ppb)	Complete absence or severely decreased cilia in 5 cases, normal microvilli composition but severe decrease of basal bodies in 5 cases, mislocalized basal bodies and attached rootlets in 3 cases	Complete absence or markedly reduced cilia in all mutant airway cells	Homozygous mutation, c.258_262dupGGCCC (p.Gln88Argfs*7) in 5 cases; c.248_252dupTGCCC (p. Gly85Cysfs*10) in 5 cases; c.263_267dup(p.Val90Serfs*5) in 2 cases; c.926delC (p.Pro309Argfs*17) in 1 case; c.961C>T (p.Gln321*) in 1 case; c.716A>G (p.His239Arg) in 1 case; Compound heterozygous mutation, c.248_252dupTGCCC (p.Gly85Cysfs*10) and c.481_482delCT (p.Leu161Glyfs*72) in 1 case
Casey et al. (2015)	Irish traveler family	2	2 siblings, males, recurrent LRTI, the elder sibling has a history of recurrent otitis media but the younger sibling does not, neither have recurrent sinusitis or situs inversus	Universally low at 30-50ppb	Nude epithelium in 1 case	N/A	Homozygous mutation, c.258_262dup(p.Gln88Argfs*8) for both
Amirav et al. (2016)	Israel	15	9 males, 6 females, age at genetic diagnosis between 6-54 years old, all were born at term, nRDS (11/14), otitis (10/15), rhinorrhea/sinusitis (13/14), bronchiectasis (13/14), 1 got lung transplantation at age 43, 1 had arrested hydrocephalus, 1 female underwent in vitro fertilization, all had chronic cough and had no situs inversus, one patient with diffuse micronodules and "tree-in-bud" sign in right lung	11 lower than 77nl/min; 3 at normal range (99, 255.75, 99nl/min respectively; 1 not available	Completely lack basal bodies and cilia but still protrude microvilli in 2 cases, mislocalized rootlets as well as microvilli in 1 case	N/A	Compound heterozygous mutation, c.258_262dupGGCCC (p.Gln88Argfs*8) and c.481_482delCT (p.Leu161Glyfs*73) in 2 cases; Homozygous mutation, c.258_262dupGGCCC (p.Gln88Argfs*8) in 6 cases; Homozygous mutation, c.638T>C (p.Leu213Pro) in 4 cases; Homozygous mutation, c.165delC (p.Gly56Alafs*38) in 1 case; Compound heterozygous mutation, c.258_262dupGGCCC (p.Gln88Argfs*8) and c.638T>C (p.Leu213Pro) in 1 case;

							Compound heterozygous mutation, c.165delC (p.Gly56Alafs*38) and c.258_262dupGGCCC (p.Gln88Argfs*8) in 1 case
Guo et al. (2017)	China	2	23 years old female and 29 years old male, both had bronchiectasis and rhinosinusitis in CT, neither had hearing problem	Both had low nNO: 3.3ppb and 31ppb, respectively	N/A	N/A	Homozygous mutation, c.248_249insGCCCG (p.Q88Rfs*8)
Shen et al. (2019)	China	1	10-year-old female, history of neonatal pneumonia, sinusitis, a brother died at 14 years of age due to lung disease, small nodules in left lung and right middle and lower lung, bronchiectasis in lung CT	27.2nL/min	N/A	N/A	Compound heterozygous mutation, c.848T>C(p.L283P) and c.262_263insGGCCCGGCCC(p.Q88Rfs*51)
Emiralioğlu et al. (2020)	Turkey	4	1 male, 3 females, age at diagnosis: 0.5-4 years old, nRDS (4/4), chronic rhinitis (3/4), recurrent sinusitis (3/4), recurrent otitis (1/4), hearing impairment (0/4), situs inversus totalis (0/4), clubbing (0/4), congenital heart defect (2/4), history of lobectomy (1/4), bronchiectasis (1/4)	5-18ppb	N/A	Hypokinetic cilia in 4 cases	Homozygous mutation, c.263_267dupAGCCC (p.Val90Serfs*6) in 2 cases; c.564_567+1delinsGCGATGCAAGCGATGCAAGCGATGCAAGCGATGA (protein variant N/A) in 2 cases
Henriques et al. (2021)	Portugal	3	Case 1: 11-year-old male, history of neonatal respiratory distress, requiring ventilatory support for 3 days and supplemental oxygen for 12 days, no laterality defects, persistent wet cough and recurrent LRTI, persistent left lower lobe collapse, left lower lobectomy at age 4, persistent mild obstructive ventilatory impairment<br>Case 2: 11-year-old female, year-round daily productive cough, nasal congestion and recurrent LRTI, fewer exacerbation after age 7, digital clubbing presented at age 5, chest radiograph showed bilateral bronchial wall thickening and atelectasis of the left lower lobe at age 4 and bilateral cylindrical bronchiectasis at age 5<br>Case 3: 11-year-old female, neonatal respiratory distress, requiring supplemental oxygen for 10 days, persistent wet cough, chronic rhinosinusitis, otitis media with effusion and conductive hearing loss, recurrent	11-137ppb (lower than the level suggestive for PCD)	Reduced or absent cilia in 3, normal ultrastructure in 2 cases	Few or absent cilia in 3 cases, uncoordinated CBP in 3 cases, normal CBF in 2 cases	Compound heterozygous mutation, c.253_257GGCCC (p.Gln88fs) and c.263_267dup(p.Val90fs) in one case;<br>Homozygous mutation, c.263_267dup (p.Val90fs) in 2 cases

			LRTI, failure to thrive, digital clubbing presented at age 4, bronchiectasis showed at age 3, tympanostomy tube insertion for twice, mild obstructive ventilatory impairment				
Guan et al. (2021)	China	3	3 males, age at diagnosis: 6.2±3.2 years old (mean±standard deviation), nRDS (2/3), bronchiectasis (1/3), atelectasis (3/3), situs inversus (0/3), asthma (2/3)	20.8 (16.5-55.8nl/min), median (interquartile range)	Oligocilia in 1 case	N/A	Homozygous mutation, c.262_c.263insGGCCC (p.Q88Rfs*8) in 1 case; c.262_c.263insGGCCCGGCCC (p.Q88Rfs*51) in 1 case; Compound heterozygous mutation, c.267_c.268insAGCCC (p.V90Sfs*6) and c.940G→T (p.E314X) in 1 case
Zhang et al. (2022)	China	1	22-year-old female, recurrent productive cough, “tree-in-bud” sign and miliary nodules in the left lower lung, bronchiectasis, bilateral maxillary and ethmoid sinusitis in CT scan, no visceral transposition	N/A	Few and short cilia	N/A	Compound heterozygous mutation, c.303C>A and c.248_252dup
Celiksoy et al. (2023)	Turkey	1	22-year-old male, respiratory distress at 5 days old, recurrent cough with sputum and a runny nose since birth, no history of recurrent otitis or sinusitis, cystic bronchiectasis in lung CT, selective IgM deficiency	N/A	N/A	CBF was 0.83±0.11Hz, short cilia with minimal or no movement	Homozygous mutation, c.493delC (p. Leu165SerfsTer33)
Wang et al. (2023)	China	1	8-year-old female, dyspnea at birth and treated with mechanical ventilation for 10 days, recurrent cough with wheeze, tubbiness thorax with clubbing finger, diffuse centrilobular nodules throughout both lungs with tree-in-bud sign, maxillary and ethmoid sinusitis on HRCT, no ciliary structure on bronchial mucosa biopsy	N/A	N/A	N/A	Compound heterozygous mutation, c.263_267dupAGCCC and c.258_262dupGGCCC
Gong et al. (2023)	China	1	9-year-and-10-month-old female, history of neonatal respiratory distress and atelectasis, recurrent cough and nasal discharge, otitis media, short stature (growth hormone deficiency)	N/A	N/A	N/A	Homozygous mutation, c.323del (p.F108Sfs*21)
Petrarca et al. (2023)	North Africa	1	2-month-old female, Neonatal distress 24 hours after birth, 3 more times of acute respiratory distress and need oxygen supplement within first year, chronic rhinitis, complete atelectasis of the left apical lobe	N/A	Poor number of cilia	N/A	Homozygous mutation, c.258_262dup (p.Gln88Argfs*8)

Alhalabi et al. (2024)	India	2	Two siblings, 17 years and 15 years old females, both experienced lower respiratory infection, chronic rhinorrhea, recurrent ear infection, chronic wet cough, progressive loss of lung function, the elder sister required lung transplantation, both had digital clubbing, bronchiectasis was detected at age 14 of the elder sister and age 7 of the young sister, the elder sister showed bilateral varicose bronchiectasis, the younger sister showed diffuse cystic bronchiectasis and “tree-in-bud” changes	Both had low nNO: 63.3ppb, 88.3ppb, respectively	N/A	N/A	Homozygous mutation, c.258_262dup (p.Gln88Argfs*8) in both cases
Xu et al. (2024)	Japan	2	5 years and 13 years old males, both were full-term, both had history of neonatal respiratory distress, both had persistent wet cough and rhinosinusitis, neither had situs inversus, one had refractory otitis media with effusion, one showed bronchiectasis at 13 years of age, one had atelectasis	N/A	Complete absence or a remarkable reduction of cilia, microtubular disorganization, residual cilia lack central pairs, mislocalized rootlets in the cytoplasm in 1 case	N/A	Compound heterozygous mutation, c.262C>T (p.Gln88Ter) and c.781delC (p.Leu261fs) in 1 case; c.262C>T (p.Gln88Ter) and c.248_252dupTGCCC (p. Gly85fs) in another

Abbreviations:
nNO: nasal nitric oxide; TEM: transmission electron microscopy; HSVA: high-speed videomicroscopy analysis; CBP: ciliary beat pattern; CBF: ciliary beat frequency; LRTI: lower respiratory tract infection; nRDS: neonatal respiratory distress syndrome; N/A: not applicable; IgM: immunoglobulin M; ppb: parts per billion
